# 

**Published:** 1873-08

**Authors:** 


### CONTENTS.

ORIGINAL COMMUNICATIONS.

Fever. By Thomas A. Cook, . . . . . . . . . . . 449
Address before the Lowndes County (Miss.) Medical Society, at the Anniversary meeting, May 29, 1873. By Drs. J. W. M. Shattuck and W. L. Lipscomb, . . . . . . . . . . . . . . 467

SELECTIONS.

Management of Infants in Hot Weather, . . . . . . . . . 475
Treatment of Psoriasis, . . . . . . . . . . . . . 478

EXTRACTS AND GLEANINGS.

A New Remedy for Colic—479. Quinine and Utero-gestation; To Bend Glass Tubes—480. A New Remedy for Syphilis; Prophylaxis in Scarlatina—481. Quinine by the Rectum; Electro-magnetic Paper—482. Phytolacca Decandra in the Treatment of Inflammation of the Mammary Glands; Bromide of Potassium in Cholera—483. The Treatment of Boils; Acute Articular Rheumatism—484. Hypodermic Treatment of Collapse; Hypodermic Injection Sulphate of Morphia in Autumnal Catarrh: Neuralgia—485. Extreme Depression and Paralysis Produced by Hydrate of Chloral; Valentine's Preparation of Meat Juice; Mosquito Netting as a Surgical Dressing—486. Bromide of Potassium and the Diseases of Dentition; Night Sweats of Phthisis; Chronic Diarrhœa—487. Calabar Bean in Constipation and Hemicrania; Carbolic Acid as a Local Anæsthesia in Boils, Whitlows, etc.; Strychnia in Amaurosis—488. Gelseminum in Gynæcology; Erysipelas; Burns—489. Hydrate of Chloral in Sclerosis of the Middle Ear ("Dry Catarrh"); Hypodermic Injection of Quinia in Heat Apoplexy; The External Application of Veratrum Viride—490. Monti on Constipation in Children; Actæa Racemosa; A New Use for Old Stockings—491. Small Pox Keratitis; Sulphuric Acid as a Prophylactic in Cholera; Radical Cure of Fistula Ani without the Use of the Knife—492. Eucalyptus as an Antiseptic; Sponge-tent in Epistaxis; Use of Placental Forceps through the Speculum; Arnica Liniment—493. Persistent Vomiting; Chilblains; Nævi Cured by Monsel's Solution Applied Externally—494. Displacement of the Uterus Treated by Electricity; Neuralgia and Sciatica Cured by Turpentine—Turpentine Pearls; Ulcers; Hops as an External Anodyne—495. Salicin in Obstinate Diarrhœa; Rules for giving Belladonna in Opium Poisoning; Chancroids; Bright's Disease—496. Podophyllin; Erysipelas; Formula for Hysteria; Cramps Attending Pregnancy; Pruritus Vulvæ; Diabetes—497. Atropia Subcutaneously in Cholera; Treatment of Diabetes Mellitus; Atropized Castor Oil in Corneal Disease; Subacute Pleurisy; For Catarrh; Stokes' Liniment; Juniper Tar Soap—488. Action of Ergot; Ice in the Treatment of Facial Neuralgia; Etherized Cod-Liver Oil; Treatment of Croup; Tincture Iodine, Colorless; Laxative Powder of Jeannel; For Chilblains; Mucilage for Office Use; Neuralgia, Gray's Powder—499.

EDITORIAL AND MISCELLANEOUS.

Our Thanks; Our Editorial Space, . . . . . . . . . 500
A Proposition, . . . . . . . . . . . . . . 301
Letter from Dr. W. T. Taylor, of Kentucky, . . . . . . . . 502
From one of our Associates, . . . . . . . . . . . 504
The Senior Editor; Demorest's Monthly; Cholera, . . . . . . 506
Bibliographical, . . . . . . . . . . . . . . 507
Scientific American; The Plantation; Scribner's Monthly; List of Cash Payments to the Record, . . . . . . . . . . . 509
In Memoriam, . . . . . . . . . . . . . . . 511

